# Advances in Polyhydroxyalkanoate Nanocarriers for Effective Drug Delivery: An Overview and Challenges

**DOI:** 10.3390/nano12010175

**Published:** 2022-01-05

**Authors:** Priyanka Prakash, Wing-Hin Lee, Ching-Yee Loo, Hau Seung Jeremy Wong, Thaigarajan Parumasivam

**Affiliations:** 1 School of Pharmaceutical Sciences, Universiti Sains Malaysia, Minden 11800, Penang, Malaysia; priyankaprakash95@outlook.com; 2 Faculty of Pharmacy and Health Sciences, Royal College of Medicine Perak, Universiti Kuala Lumpur (RCMP UniKL), Ipoh 30450, Perak, Malaysia; whlee@unikl.edu.my (W.-H.L.); cyloo@unikl.edu.my (C.-Y.L.); 3 School of Biological Sciences, Universiti Sains Malaysia, Minden 11800, Penang, Malaysia; jeremywong@student.usm.my

**Keywords:** drug delivery, polyhydroxyalkanoates, nanocarrier, nanotechnology, challenges

## Abstract

Polyhydroxyalkanoates (PHAs) are natural polymers produced under specific conditions by certain organisms, primarily bacteria, as a source of energy. These up-and-coming bioplastics are an undeniable asset in enhancing the effectiveness of drug delivery systems, which demand characteristics like non-immunogenicity, a sustained and controlled drug release, targeted delivery, as well as a high drug loading capacity. Given their biocompatibility, biodegradability, modifiability, and compatibility with hydrophobic drugs, PHAs often provide a superior alternative to free drug therapy or treatments using other polymeric nanocarriers. The many formulation methods of existing PHA nanocarriers, such as emulsion solvent evaporation, nanoprecipitation, dialysis, and in situ polymerization, are explained in this review. Due to their flexibility that allows for a vessel tailormade to its intended application, PHA nanocarriers have found their place in diverse therapy options like anticancer and anti-infective treatments, which are among the applications of PHA nanocarriers discussed in this article. Despite their many positive attributes, the advancement of PHA nanocarriers to clinical trials of drug delivery applications has been stunted due to the polymers’ natural hydrophobicity, controversial production materials, and high production costs, among others. These challenges are explored in this review, alongside their existing solutions and alternatives.

## 1. Introduction

In today’s modern society, the leaps and bounds taken by medical advancements are no stranger to humankind. That being said, drug delivery systems (DDSs), otherwise defined as pharmaceutical apparatus or formulations that aid in the sustained, targeted release of a therapeutic agent [1], have also become increasingly efficient, with the utilization of modern technology and techniques to enhance the transport and release of drugs once administered to patients. However, despite best efforts, side effects can crop up because of the unsuitability of the drug and/or its administration route, as well as the patient’s immune response. To avoid undesirable side effects and enhance healing, it is essential that the drug concentration be sustained at its optimal therapeutic range, which would involve a delicate blend of an appropriate number of doses, a suitable administration route, a controlled rate of drug release, as well as the right type of DDS [2]. Given these stringent requirements to ensure effective treatment, it is crucial that promising options for DDSs be explored thoroughly, and one such candidate is the nanocarrier.

Nanocarriers are a relatively new delivery system characterized by therapeutic particles with a size of less than 500 nm [3,4,5]. Their high surface-area-to-volume ratio gives rise to many desirable characteristics such as enhanced biodistribution, increased stability, and improved bioactivity and pharmacokinetics [6]. Their nanosize is also particularly useful as it allows the nanocarriers more freedom to traverse the human body compared to DDSs of larger size [7]. Nanocarriers can also remain in the circulatory system for an extended period, which not only eases drug release according to their doses, but also decreases unwanted side effects [8]. In order to achieve targeted delivery while also bypassing the biological barriers in the human body that might denature or degrade the drug, nanocarriers have to be designed appropriately using a material with suitable properties [9], and this is where polyhydroxyalkanoates come into the picture.

**Figure 1:**
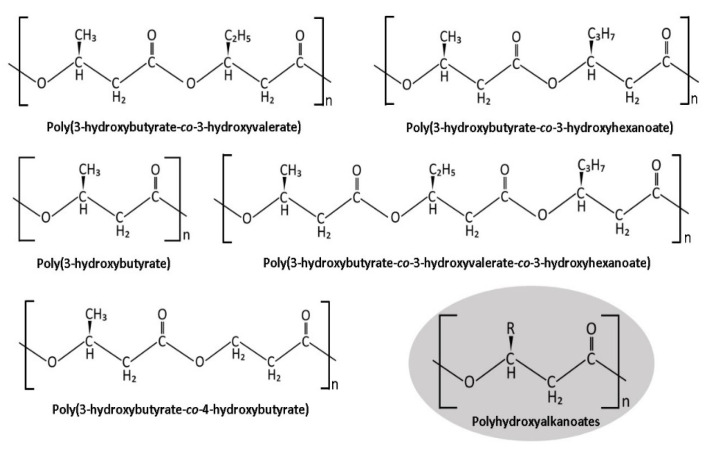
Chemical structures of P(3HB-*co*-3HV), P(3HB-*co*-3HHx), P(3HB), P(3HB-*co*-3HV-*co*-3HHx), P(3HB-*co*-4HB), and PHA.

Polyhydroxyalkanoates (PHAs) are biopolymers that are naturally produced by certain types of bacteria and other organisms as an energy source during periods of unstable growth. When certain nutrients become limited and carbon is excessive, PHA granules are synthesized by bacterial cells for long-term survival. PHA-producing microorganisms include *Cupriavidus necator*, *Chromatium vinosum*, and *Pseudomonas aeruginosa* [10]. PHA consists mostly of short-chain-length hydroxyalkanoic acids (scl-PHAs) with monomers of three to five carbon atoms and medium-chain-length hydroxyalkanoic acids (mcl-PHAs) with monomers of six to fourteen carbon atoms. A less common class of PHA is long-chain-length hydroxyalkanoic acids (lcl-PHAs), which have monomers of more than fourteen carbon atoms [11]. PHAs as shown in Figure 1 are popular in the field of nanomedicine, given their high loading capacity, biocompatibility, lack of toxicity, and biodegradability [12]. PHAs triumph over other bioplastics in the medical field because their monomers 3-hydroxybutyric acid (3HB) and 4-hydroxybutyric acid (4HB) are recognized by the human body as degradation products, conveniently resulting in their swift, natural removal from the body [13]. Additionally, PHA-based DDSs have proven to be highly effective because they can achieve a targeted drug delivery with the aid of targeting ligands and can also provide a controlled release of the incorporated drugs [14]. Hence, it is no surprise that PHAs have been utilized in DDSs for cancer therapy and other diverse applications [15].

Despite having many redeeming qualities, certain characteristics of PHAs may discourage their use in the medical field. Some of these include a high hydrophobicity, low thermal stability, and a slow degradation rate [16]. However, their most notable drawback lies in their high production costs, which in turn hinder the commercialization of PHAs. This is also the reason PHAs tend to be overlooked for usage in medical applications despite their ideal properties. Their high price is the consequence of a need for a large amount of substrates with high purity, as well as their labor-intensive production and downstream processing [17]. To amend this, researchers have taken to exploring more sustainable alternatives such as using waste materials as carbon sources, and replacing chemical extraction methods with biological ones. In time, PHAs will undoubtedly claim their place at the forefront of superior biomaterials, which will encourage their advancement into clinical trials in the development of nanocarriers as therapeutic agents.

This review explores current formulation strategies and applications of PHA nanocarriers in DDSs alongside their pharmacokinetics, pharmacodynamics, and other key findings. The article also looks into the challenges of utilizing PHAs in nanomedicine, as well as existing solutions to them.

## 2. Formulation of PHA Nanocarriers

In the past decade, PHA-based nanoparticles as drug carriers have garnered significant attention for treating various diseases, owing to their potential to improve existing drug delivery systems via the design of novel dosage forms. Such formulations could have a better treatment outcome than conventional therapy due to their promising physicochemical properties as mentioned earlier [18], including (i) the ability to overcome the solubility of hydrophobic drugs, (ii) being readily manipulated for active targeting, (iii) the stabilization of chemotherapeutic agents, (iv) full biocompatibility and non-immunogenicity, and (v) superior pharmacokinetics and pharmacodynamics compared to free drug therapy [19,20,21,22,23]. However, US Food Drug Administration (FDA)-approved PHA-based nanomedicines for treatment are unavailable. PHA and nanotechnology-based therapies are still in the experimental stages, and clinical trials are significantly lagging.

**Figure 2:**
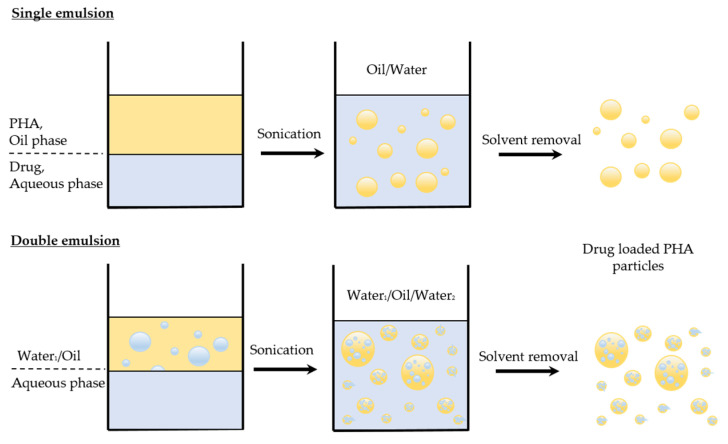
The emulsion solvent evaporation method to produce PHA nanoparticles. The drug is either dissolved in the oil phase with the PHA (oil/water, single emulsion) or emulsified in the oil phase (water_1_/oil/water_2_, double emulsion) and then further emulsified in the continuous water phase. This is followed by solvent evaporation and washing with distilled water.

PHA-based nanoparticles have been explored for the encapsulation of a wide range of therapeutic agents, including anticancer agents, antibiotics, hormones, and vaccines [24,25]. Different formulation strategies have been explored to produce PHA nanoparticles, including emulsion solvent evaporation techniques (i.e., oil-in-water (O/W) single emulsion, and water-in-oil-in-water (W/O/W) double emulsion), nanoprecipitation, dialysis, and in situ polymerization techniques [26,27,28,29] as summarized in Table 1. The selection of the formulation approach mainly depends on the intended particle size, morphology, and solubility of the target drug and the polymer. The emulsion solvent evaporation method has been utilized the most to produce PHA-based nanoparticles because this technique eases control of the processing parameters and allows encapsulation of both hydrophobic and hydrophilic drugs [30]. Generally, the drug is either dissolved or emulsified in the oil phase, then further emulsified in the continuous aqueous phase, as shown in Figure 2. This is followed by solvent evaporation to allow the hardening of the particles. The particles are then washed with distilled water, collected via centrifugation, and freeze-dried for long-term storage [31].

For instance, in a recent study, Hu and co-workers produced poly(3-hydroxybutyrate-*co*-3-hydroxyvalerate-*co*-3-hydroxyhexanoate) (PHBVHHx) nanoparticles loaded with immunosuppressant drug azathioprine for the potential treatment of systemic lupus erythematosus using the emulsion solvent technique. The particles not only had acceptable toxicity and slow clearance from kidneys, but they also exhibited a higher therapeutic effect compared to polylactic acid (PLA) nanoparticles when tested in a murine systemic lupus erythematosus model [32]. Similarly, Xiong et al. reported that poly(3-hydroxybutyrate) (PHB) and poly(3-hydroxybutyrate-*co*-12 mol% 3-hydroxyhexanoate) (PHBHHx) nanoparticles with a size range of 160-250 nm loaded with rhodamine B isothiocyanate (a lipid-soluble dye) deeply penetrated macrophages and prolonged drug release to about 20 days compared to its PLA counterpart which took about 15 days. This proves that PHA-based nano-systems can provide a slower drug release with an enhanced therapeutic index compared to PLA nanoparticles that have been well-studied as a drug control release system.

The surface of PHA nanoparticles can also be functionalized to enhance the localization of the particles in the vicinity of the cells. For example, curcumin-loaded and conjugated with targeting ligand concanavalin A in poly(3-hydroxybutyrate-*co*-3-hydroxyhexanoate) (PHBHHx) nanoparticles (average size of 228 ± 5 nm) showed enhanced cellular uptake and apoptotic activity in breast cancer cells compared to the non-functionalized nanoparticles [33]. A similar higher in vitro cellular uptake was reported with etoposide (an antineoplastic agent)-loaded poly(3-hydroxybutyrate-*co*-3-hydroxyhexanoate) (PHBHHx) nanoparticles functionalized with folic acid in HeLa cells [34]. As anticipated, P(3HV-*co*-4HB)-*b*-mPEG, an amphiphilic poly(3-hydroxyvalerate-*co*-4-hydroxybutyrate) and polyethylene glycol nanoparticle, enhanced the apoptotic activity of the encapsulated cisplatin compared to the free drug-treated group using a DU145 prostate cancer cell line [35]. Thus, these findings merit that PHA nanoparticles, like other polymeric nanoparticles, are suitable for targeted drug delivery systems when conjugated with targeting moieties.

On the other hand, a common drawback of PHB nano-delivery systems, especially those using scl-PHB, is the rapid release of the encapsulated drugs. However, this problem can be mitigated by conjugating the drug molecule to the PHB polymer to slow the drug release [36]. Additionally, the rapid release of scl-PHB can be altered by converting it into PHB glyoxylate via ozonolysis for a slow release of drugs possessing primary amine groups [37]. Hence, the development of PHA-drug conjugates opens a new window for novel slow-release drug delivery therapies.

Similar to other colloidal systems, PHA nanoparticles also suffer the disadvantage of poor drug loading [38]. For instance, a drug loading of less than 30% has been reported in various studies [30,35,39,40,41,42,43]. As a solution, the strategies reported for other polymers such as PLGA can be used as a guideline to increase the drug loading of PHA-based nanoparticles. An extensive review of strategies to increase the drug loading of polymeric particles can be found at [30,44].

**Table 1:** Key findings of various polyhydroxyalkanoate (PHA) nanoparticle formulations.

Polymer	Drug	Size (nm)	Drug Loading (%)	Formulation Method	Key Findings	Ref.
Poly(3-R-hydroxyalkanoate)	Calcein and Nile red	155	-	Nanoprecipitation	Unsaturated PHA is suitable to make controlled release nanomedicine.	[45]
Poly(3-hydroxybutyrate-*co*-3-hydroxyvalerate-*co*-3-hydroxyhexanoate) (PHBVHHx)	Azathioprine	95.7	-	Modified emulsion	The particles have acceptable toxicity and slow clearance from kidneys, with a higher therapeutic effect than polylactic acid (PLA) nanoparticles when tested in a murine systemic lupus erythematosus model.	[32]
Poly(3-hydroxybutyrate-*co*-3-hydroxyhexanoate) (PHBHHx)	Curcumin	273 ± 84	15–30	Solvent evaporation	Lyophilization is suitable for preserving the nanoparticles at 4 °C. The particles had high apoptotic activity and localization into MDA-MB-231 cells.	[33]
Poly(3-hydroxybutyrate-*co*-3-hydroxyhexanoate) (PHBHHX)	Etoposide	180–1500	2.92–8.77	Modified solvent evaporation	Folic acid-conjugated nanoparticles have higher selectivity to cancer cells than fibroblast cells.	[34]
Poly(3-hydroxyvalerate-*co*-4-hydroxybutyrate)	Cisplatin	155 ± 5	9.58 ± 1	Emulsification–solvent evaporation	Cisplatin-loaded PHA nanoparticles accumulated in tumour cells and showed significant tumour deterioration compared to free drug treatment.	[35]
Poly(3-hydroxybutyrate-*co*-3-hydroxyvalerate) (PHBV)	Nile red	166–426	-	Oil-in-water emulsion	The nanoparticles penetrated the skin of the BALB/c mouse model without adverse effects.	[46]
Poly (3-hydroxybutyrate-*co*-12 mol% 3-hydroxyhexanoate) (PHBHHx) and Poly (3-hydroxybutyrate-*co*-5 mol% 3-hydroxyhexanoate) (PHBV)	TGX-221	195–220	8.5–8.8	Modified emulsification/solvent diffusion	The encapsulation of TGX-221 in PHA nanoparticles could mitigate the poor bioavailability and limited in vivo half-life of the TGX-221.	[39]
Poly-3-hydroxybutyrate-*co*-5 mol% 3-hydroxyvalerate (PHBV-S), poly-3-hydroxybutyrate-*co*-11 mol% 3-hydroxyvalerate (PHBV-11) and poly-3-hydroxybutyrate-*co*-15 mol% 3-hydroxyvalerate (PHBV-15)	Ellipticine	184–283	-	Modified emulsification–solvent evaporation	The particles showed no inhibition of the A549 cancer cell line at various tested concentrations (i.e., 250.0, 62.5, and 15.6 μg/mL).	[47]
Poly(3-hydroxybutyrate-*co*-3-hydroxyhexanoate) (PHBHHx)	Rapamycin	200	8.47–8.52	Emulsification–solvent evaporation	The particles showed an efficient entrapment of 91.9% and a sustained release of rapamycin for almost 10 days. Cellular uptake of PEG200 end-capped nanoparticles was significantly higher than that of non-PEG nanoparticles in a human prostate cancer cell line and a murine macrophage cell line.	[40]
Polyhydroxybutyrate, poly(hydroxybutyrate-*co*-hydroxyvalerate) P(HB-HV) with 12 and 50% HV	5,10,15,20-Tetrakis(4-hydroxy-phenyl)-21*H*, 23*H*-porphine	169.0–211.2	0.91–46.64	Emulsification-diffusion	The particles showed a concentration and time-dependent photocytotoxicity in a human colon adenocarcinoma cell line.	[48]
Poly(3-hydroxyoctanoate-*co*-3-hydroxyhexanoate) (PHOHHx)	-	44–90	-	Dialysis	A series of diblock copolymers of PHOHHx with poly(ethylene glycol) (PEG) were synthesized using “click” chemistry and assembled into micelles for drug delivery.	[29]
Poly(3-hydroxybutyrate-*co*-3-hydroxyvalerate) P(3HB-*co*-3HV) or poly(3-hydroxybutyrate-*co*-4-hydroxybutyrate) P(3HB-*co*-4HB)	Thymoquinone	112–162	-	Modified emulsification–solvent evaporation	The chemical combination of PHA copolymers and mPEG-based nanoparticles was nontoxic and biocompatible to prenatal rat neuronal hippocampal and NIH/3T3 fibroblast cells in vitro.	[49]
Polyhydroxybutyrate (PHB)	NuBCP-9	126 ± 8	-	Double emulsion solvent evaporation	PEG-conjugated PHB nanoparticles showed a sustained release of NuBCP-9 for up to 26 days and efficient cellular uptake in a time-dependent manner in MCF-7 cells. A 90% tumour regression was seen when particles were administered intraperitoneally twice a week for three weeks in an Ehrlich syngeneic mouse model.	[50]
Polyhydroxybutyrate (PHB)	Nile red	-	-	Oil-in-water emulsion solvent evaporation	PHB functionalized with tumour-specific ligand nanoparticles showed a specific affinity to MDA-MB-231 breast cancer cells.	[51]
Poly(3-hydroxybutyrate-*co*-3-hydroxyhexanoate) (PHBHHx)	Rhodamine B isothiocyanate	100–200	-	Oil-in-water emulsion	The recombinant human a1-acid glycoprotein or recombinant human epidermal growth factor functionalized nanoparticles were taken up by macrophages and hepatocellular carcinoma cells.	[52]
Poly(3-hydroxybutyrate-*co*-3-hydroxyvalerate) (PHBV)	-	133–300	-	Miniemulsification and emulsion/solvent evaporation	An increase of the polymer concentration led to a larger particle size due to a change in viscosity.	[53]
Poly([R,S]-3-hydroxybutyrate) (PHB)	Doxorubicin and sorafenib	199.3–250.5	2.6–8.4	Nanoprecipitation	Co-encapsulation of dual anticancer drugs was achieved. A sustained and faster drug release was observed for doxorubicin and sorafenib, respectively.	[41]
Poly(hydroxioctanoate-*co*-hexanoate)	-	63 ± 4	-	Emulsion-solvent evaporation	The particles interacted with pulmonary surfactant proteins and lipids, which may limit the use of PHA for pulmonary drug delivery.	[54]
Polyhydroxyalkanoate (PHA)	-	145–159	-	Oil-in-water emulsion	The PHA nanoparticles showed antibacterial activity against *S. aureus*, *S. pneumoniae*, *E. coli*, *K. pneumoniae,* and *P. aeruginosa*.	[55]
Poly(3-hydroxybutyrate-*co*-3-hydroxyoctanoate) (P(HB-HO))	Doxorubicin	240	29.6	Water-in-oil-in-water solvent extraction/evaporation	Doxorubicin-loaded folate-mediated nanoparticles were readily internalized by HeLa cells in vitro.	[42]
Polyhydroxybutyrates (PHB)	Concanavalin-A and etoposide	239.43 ± 5.25	-	Multi-emulsion	Iron oxide particles were successfully coated with PHB. The cytotoxicity of these magnetic PHB particles were reported against cancer and non-cancer cells.	[56]
Poly(hydroxybutyrate-*co*-hydroxyvalerate) (PHBV)	Fingolimod	250	0–22.5	Single and double evaporation	The optimal preparation of PHBV nanoparticles required a polymer concentration of 1.32%, a PVA concentration of 0.42%, and 5 mg of the drug.	[43]

Note: ‘-’ indicates the value was not reported.

## 3. Applications of PHA Nanocarriers

### 3.1. Treatment of Cancer

PHA nanoparticles have been suggested as an alternative to conventional polymeric nanoparticles, namely poly(D,L-lactide-*co*-glycolic) acid (PLGA)-based nanocarriers, for the delivery of hydrophobic anticancer agents for therapeutic purposes [41]. Di Mascolo et al. demonstrated that the cytotoxic potential of docetaxel (DCT)-loaded poly(3-hydroxybutyrate) P(3HB) nanoparticles (DCT-P3HB-NPs) against U-87 MG cells was comparable to DCT-loaded PLGA-NPs [57]. However, owing to the hydrophobic characteristic of P(3HB) compared to PLGA, it allowed higher drug loading (by twofold) and a slower drug release [57]. Additionally, the degradation product of P(3HB) is 3-hydroxybutyric acid which is non-toxic in blood plasma compared to the acidic degradation products of PLGA that could lead to inflammation.

One of the drawbacks of PHAs which hampers their application as a carrier is their limited solubility due to their hydrophobicity. An amphiphilic PHB copolymer incorporating the hydrophilic polyethylene glycol (PEG) was synthesized via transesterification reactions to overcome PHB’s hydrophobicity [58]. This modification also prevented the opsonization and phagocytic clearance of PHB as well as enhanced the cellular uptake [59]. Subsequently, the authors evaluated the effectiveness of PHB-*co*-PEG copolymer nanoparticles loaded with an antisense oligonucleotide (ASN) against breast (MDA-MB-231) and lung (A549) cancer cell lines [58]. The viabilities of A549 and MDA-MB-231 cells exposed to 200 mg/mL of ASN-loaded PHB copolymeric nanoparticles were 49.89% and 35.34%, respectively. The authors demonstrated that the use of PHB copolymeric nanoparticles protected ASN from enzymatic degradation as well as enhanced its cellular internalization [58]. When conferring hydrophilic properties onto PHA, the molecular weight of PEG is also important to control the loading efficacy and drug release rate [60]. PEG can also form a stable complex with enzymes, which can then be immobilized onto PHB nanoparticles for improved anti-proliferative activity against cancer cells. In a study by Pandian et al., L-glutaminase/PEGylated-PHB nanoparticles enhanced the inhibition of HeLa cell proliferation in vitro via glutamine deprivation [61]. A significant increase in DNA damage, ROS production, and caspase-3 levels was in line with the successful delivery of L-glutaminase using PEGylated-PHB nanoparticles [61].

Besides that, the functionalization of O-carboxymethyl chitosan (CMCh) onto PHB nanoparticles during synthesis was another approach to overcome its hydrophobicity and solubility issues [62]. The etoposide-loaded CMCh-PHB nanoparticles (ETP-CMCh-PHB NPs) stabilized with polyvinyl alcohol were negatively charged and displayed a sustained release behaviour. When treated with 100 µg/mL of ETP-CMCh-PHB NPs and blank CMCh/PHB, the viability of MCF-7 cells was 61.13% and 101.8%, respectively [62].

Masood et al. studied the effects of different hydroxyvalerate (3HV) units within a PHB copolymer to determine its suitability as a nanocarrier for anticancer agents [47]. Irrespective of the 3HV monomer content, the ellipticine-loaded PHB-*co*-3HV nanoparticles demonstrated no statistical differences in their cytotoxic effect against A549 cancer cells. Nonetheless, these nanoparticles incited a higher cell inhibition of almost twofold compared to free ellipticine [47]. As the degradation rate of PHB-*co*-3HV nanoparticles is slow, the concentration of the released drug from the polymeric nanoparticle is expected to be low. As demonstrated by Vilos et al., less than 1% of paclitaxel (PTX) was released over a 5-day period in physiological buffered media (pH 7.4), which suggests that a highly specific targeted application be considered in the future for this nanoparticle [63]. This finding is in agreement with previous work whereby a sustained drug release profile that lasted for more than 20 days was observed for PHB and poly(3-hydroxybutyrate-*co*-3-hydroxyhexanoate) (PHB-*co*-HHx) nanoparticles [64]. To assess the clinical relevance of fabricated PTX-loaded PHB-*co*-3HV NPs, six primary cell cultures obtained from patients undergoing treatment for stage IIIc papillary serous ovarian cancer were treated with 5 µM of PTX-loaded PHB-*co*-3HV NPs and PTX alone for 48 h. Significant cell deaths were noted for all primary cell cultures, although PTX alone was more cytotoxic than PTX-loaded PHB-*co*-3HV NPs [63].

To achieve targeted delivery, Kilicay et al. demonstrated that ETP-loaded folic acid (FA)-functionalized poly(3-hydroxybutyrate-*co*-3-hydroxyhexanoate) (ETP/FA-PHB-*co*-HHX-NPs) were more cytotoxic against HeLa cells compared to non-FA conjugated nanoparticles (ETP-PHB-*co*-HHX-NPs). Cell deaths of 44.2% and 30.1% were observed for ETP/FA-PHB-*co*-HHX-NPs and ETP-PHB-*co*-HHX-NPs when treated with equal drug concentrations [34]. This observation could be due to the selective targeting of folate ligands to cancer cells with overexpressed folic acid receptors. In another study, doxorubicin (DOX)-loaded FA-conjugated poly(3-hydroxybutyrate-*co*-3-hydroxyoctanoate) [P(HB-*co*-HO)] nanoparticles (DOX/FA PHB-*co*-HO-NPs) were significantly more cytotoxic towards HeLa cells compared to non-FA NPs. The IC_50_ of DOX/FA PHB-*co*-HO-NPs and non-FA NPs were 0.87 µM and 27.53 µM, respectively, which demonstrated that the potency of killing HeLa cells increased thirtyfold when an active targeting ligand was added [42]. A prolonged drug release pattern was noticed whereby almost 50% of DOX was released after 5 days. 

In another study, Sasikumar and Ayyasamy also observed that the release behaviour of DOX encapsulated within PHB nanoparticles using a nanoprecipitation technique followed a slow, sustained release curve [65]. Owing to the sustained DOX release, selective cancer targeting, and higher internalization rate, the reduction of tumour volume for HeLa bearing BALB/c nude mice was comparably higher for DOX/FA PHB-*co*-HO-NPs (final tumour volume: 178.91 ± 17.43 mm^3^) compared to a normal saline group (542.58 ± 45.19 mm^3^) [42].

A genetically modified PHA synthase co-expressed with a PHB chain fused with a specific ligand (Cys–Asp–Cys–Arg–Gly–Asp–Cys–Phe–Cys, RGD4C) was conjugated onto the surface of PHB nanoparticles to provide the nanoformulation with a targeted delivery towards cancer cells [51,66]. The authors ligated the chemically synthesized oligonucleotides encoding the RGD4C peptide upstream of the phaC gene to express the PHA chain fused with the RGD4C ligand. The functionalized PHB nanoparticle displayed a specific affinity towards MDA-MB-231 breast cancer cells, thus confirming the successful surface modification of a PHB nanoparticle with an active ligand [51]. In another study, phaP-fused human a1-acid glycoprotein (hAGP) or human epidermal growth factor (hEGF) ligands were attached to the surface of PHB-*co*-3HHx nanoparticles loaded with RBITC as a model drug, and were then evaluated in vitro against macrophage and hepatocellular carcinoma cells (Bel7402) [52]. Strong fluorescence signals were detected in macrophage cells after treatment with rhAGP–phaP–PHA nanoparticles, indicating that mannose-receptor mediated endocytosis was achieved with ligand-targeted recognition. Similarly, for hepatocellular carcinoma cell Bel7402 with rich EGF receptors, the in vivo delivery of nanoparticles to tumour cell-bearing mice revealed the specific recognition of hEGF–phaP–PHA nanoparticles towards tumour sites with little internalization in lung and liver tissues [52].

The use of phosphoinositide-3-kinase (PI3K) and mTOR inhibitors represent a promising approach in cancer therapy. The P13Ks belong to the family of lipid kinases that have crucial regulatory functions in cellular processes such as cell growth and survival [67]. Meanwhile, mTOR is a serine and threonine kinase that acts as sensor for energy, nutrients, and redox in metabolisms [68]. In cancer particularly, the P13K-AKT-mTOR pathway is often dysregulated and abnormally activated in human cancers in several mechanisms involving receptor tyrosine kinases (RTKs), Akt/PKB, tensin homolog (PTEN), MTOR, and other oncogene suppressor genes [69,70,71,72]. Several studies have demonstrated that TGX-221, a PI3K p110β selective inhibitor, is capable of inhibiting the growth of glioblastoma cells [73,74], prostate cancer [75], and PTEN-deficient cancer cell lines [76]. Even though it has been used as a p110β-selective inhibitor, the clinical trial outcomes were unsatisfactory owing to its low solubility and short half-life [39]. In a study by Lu et al., the encapsulation of TGX-221 in PHA nanoparticles significantly blocked the growth of PC3, BT-474, and HCT-116 cells compared to free TGX-221 [39]. The authors also showed that drug release could be altered with the incorporation of different PHA monomers depending on the crystallinity of the polymer. For instance, about 76% of drug was released within 32 h using a PHB-*co*-HHx nanoparticle compared to a 42% release for a PHB homopolymer nanoparticle [39].

In another study, a PEG200-end-capped PHBHHx (PHBHHxPEG) nanoparticle, a novel hybrid copolymer, was prepared via an emulsification–solvent evaporation method [40]. These nanoparticles, which have an average diameter of 200 nm, were loaded with rapamycin which is a natural inhibitor of mammalian target of rapamycin (mTOR) [40]. Like other studies, the hydrophobicity of the polymeric material caused the nanoparticles to be degraded at a slower rate. Therefore, the sustained release of rapamycin was achieved for almost 10 days. An almost 50% cell inhibition of prostate cancer cells was noted when the cells were treated with 100 µg/mL rapamycin-loaded PHBHHxPEG nanoparticles for 24 h. The anti-proliferation effect for rapamycin-loaded PHBHHxPEG nanoparticles were also stronger compared to free rapamycin [40].

PHA nanoparticles were also employed to encapsulate a photosensitizer compound (PS) for photodynamic therapy (PDT) in the treatment of cancer. PDT involves the combination of light and a PS to destroy abnormal tissues while exerting negligible damage towards surrounding healthy cells [77]. Similar to many other hydrophobic molecules, PS compounds suffer from stability issues due to their limited solubility in aqueous media. As demonstrated by Pramual et al., the delivery of 5,10,15,20-tetrakis(4-hydroxy-phenyl)-21H, 23H-porphine (pTHPP) using PHB-*co*-HV nanoparticles increased the in vitro photocytotoxicity against HT-29 colon cancer cells with respect to incubation time and drug concentration [48]. Cell death of up to 94% was noted when the cancer cells were exposed to 8 μg/mL of pTHPP-loaded PHB-*co*-HV nanoparticles. The faster photocytotoxicity effect compared to pTHPP-loaded NPs was probably due to a higher passive diffusion of free DMSO-solubilized pTHPP molecules [48].

PHB-coated magnetic nanoparticles were prepared via co-precipitation of iron salts (Fe^2+^ and Fe^3+^), and PHB molecules that were physically loaded with doxorubicin (DOX) were at least 2.5 times more cytotoxic against DOX-resistant MCF-7 cells compared to the free drug [78]. Drugs were released at a higher concentration at acidic conditions, mimicking the endosomal pH rather than physiological conditions (pH 7.2). Approximately 50% of the DOX was released at pH 4.5 after 9 h of incubation. In comparison, only 35% of the drugs were released at pH 7.2 [78]. In another study, similar results were found whereby ETP-loaded PHB-coated magnetic nanoparticles displayed significantly higher cytotoxic effects against HeLa cell lines compared to non-magnetic PHB nanoparticles [56]. A combined delivery of siRNA and ETP-loaded PHB-coated magnetic nanoparticles effectively downregulated the expression of multidrug resistance-associated protein 1 (MRP-1) in MCF-7/1000-etoposide resistance cells [79].

The delivery of microRNAs (miRNAs) to regulate the expression of pivotal genes involved in tumorigenesis and progression is another strategy employed in cancer treatment. For the treatment of prostate cancer, miR-124 is designed to modulate the expression of carnitine palmitoyltransferase 1A (CPT1A), which impairs the ability of cancer cells to metabolize lipid substrates. Recently, cationic polyethyleneimine-functionalized PHB nanoparticles (PEI/PHB-NPs) were utilized as a carrier to deliver miR-124 into prostate cancer cells (PC3) [80]. The delivered miR-124 interfered with the expression of CPT1A and reduced malignant cell functionality (i.e., proliferation, motility, and colony formation) [80]. Meanwhile, the delivery of miR-128 using PHB-*co*-PEI nanoparticles led to a 24.5% cell death in U87 glioblastoma cells [81], which was in accord with other findings showing that miR-128 expressions selectively downregulated glioblastoma cells compared to normal brain cells [82,83].

### 3.2. Treatment of Infectious Diseases

Many studies have explored the suitability of non-woven PHB nanofibers incorporated with various antibiotics (gentamicin sulfate, kanamycin sulphate, levofloxacin) prepared via electrospinning for antibacterial and biomedical applications [84,85,86]. The nanofibers prepared via electrospinning, an electrostatic fiber fabrication technique, are usually within the size range of 10–10,000 nm and have improved mechanical properties [87]. In addition to that, electrospun nanofibers can mimic nanoscale properties as well as stimulate the functions of a native extracellular matrix [86]. In vitro data of antibiotic-loaded PHB nanofibers demonstrated promising antibacterial activity based on good inhibition zones against *Micrococcus luteus, Serratia marcescens*, *Escherichia coli* [85], and *Staphylococcus aureus* [86]. Nanocomposites of PHBV, nanodiamond (nD), and nanohydroxyapatite (nHA) prepared using the injection molding technique enabled the sustained release of antibiotics for 22 days. The antibiotics were still active even after being exposed to high heat (178 °C) during the molding process [88].

However, with the emergence of antibiotic resistance, much attention has been diverted to the search for alternative antimicrobial agents such as metal-based agents and natural products [89,90,91,92,93,94,95,96,97]. Mukheem and groups proposed 2D nanomaterials (graphene, silver, boron nitride, molybdenum disulfide) as alternatives to antibiotics for the treatment of infections [92,93,94]. Graphene-decorated silver nanoparticles (GAg) incorporated into nanofibers of P(HB-*co*-HHx) were more effective compared to PHA/graphene oxide in killing *S. aureus* and *E. coli* [94]. Similarly, hexagonal boron nitride encapsulated within PHA/chitosan nanocomposites [92] and two-dimensional molybdenum disulfide (2D MoS2) nanoparticles [93] showed significant antibacterial activity against multidrug-resistant *E. coli* and methicillin-resistant *S. aureus* (MRSA).

Xing et al. reported the in vitro antibacterial activity of P(HB-*co*-HV) nanofibers loaded with silver nanoparticles (AgNPs) with a diameter of 5–13 nm [98]. The authors showed that of P(HB-*co*-HV) nanofibers had negligible antibacterial activity against *Staphylococcus aureus* and *Klebsiella pneumoniae*. However, the growth of both bacteria was completed inhibited for the AgNPs containing the nanofibers [98]. AgNPs embedded onto electrospun PHB nanocomposites were also virucidal against the murine norovirus (MNV) while still maintaining the nanocomposite’s optimal properties [99]. These findings implied that a customized application for packaging and contact surface industries for the eradication of viral and bacterial contamination could be possible with the proposed formulation. The compounding of a PHA nanocomposite with long alkyl chain quaternary salt (LAQ)-functionalized graphene oxide (GO-g-LAQ) improved the applications of PHA as a packaging material as denoted with a reduced oxygen permeation. Additionally, the PHA/GO-g-LAQ nanocomposites exhibited 99.9% antibacterial activity against Gram-negative and Gram-positive bacteria [97].

In a recent study, the incorporation of nitric oxide (NO) donor S-nitrosoglutathione (GSNO) onto PHB/PLA nanofibers exhibited dual antibacterial and anti-thrombotic activity [100]. The NO released from nanofibers was effective in reducing the number of attached *S. aureus* cells and platelet adhesion by approximately 80% and 65%, respectively [100]. Li et al. developed a stimuli-triggered biocide-loaded PHA-based nanofiber with a core-shell structure [101]. This formulation was designed to prevent undesirable biocide release in a physiological environment in the absence of bacteria [101]. Moreover, the authors demonstrated that the core-shell PHA-based nanofibers effectively released biocide in the presence of *P. aeruginosa*, resulting in the attenuation of bacterial growth [101].

Besides being a carrier of antimicrobial agents, a PHA nanocomposite was also developed as a biosensor for the quantitative detection of artemisinin in body fluids [95]. Artemisinin is established in the treatment of malaria and other diseases caused by highly resistant microorganisms. The PHA/gold nanoparticle mounted onto an indium-tin oxide glass plate was surface absorbed with a horseradish peroxidase enzyme to monitor the electro-catalytic reduction of artemisinin. The sensitivity and detection of this analytical method were as low as 3.5 ng/mL, which was much effective compared to other quantitative detection methods such as high-performance liquid chromatography (HPLC) and gas chromatography (GC) [95].

### 3.3. Other Applications

Peng et al. explored the use of a novel PHB nanoparticle loaded with recombinant human BMP-2 and an amphiphilic phospholipid (BPC-PHB NP) for long-term osteogenic differentiation [102]. The formulation achieved both rapid-acting and long-lasting actions of osteogenic differentiation as seen with the short initial burst release in the first 24 h followed by a steady increase of BMP-2 release for up to 20 days [102]. A novel block copolymer of PHB and PEG conjugated with deoxycholic acid was prepared via transesterification and enhanced cellular internalization and intestinal permeability. Therefore, Chaturvedi et al. attempted to develop an insulin formulation with improved oral bioavailability using this nanocomposite as the delivery vehicle [103]. Extended serum levels of insulin after oral administration were observed for insulin-loaded DOCA-PHB-PEG NPs, which is approximately 12% of the relative bioavailability [103].

## 4. Challenges and Author’s Perspective

Biocompatibility and cytotoxicity are major concerns for PHA applications as medical tools. Although the biocompatibility of PHAs is well-understood, the cytotoxicity should be minimized with caution by ensuring the purity of PHA employed in medical tool development. Repeated dissolving and precipitation of PHA in downstream processes of microbial PHA production systems are needed to guarantee the high purity of PHA [104]. However, residual organic solvents such as chloroform for PHA extraction and methanol for PHA precipitation could be a health threat if not removed completely prior to in vivo applications. The cytotoxicity of chloroform is attributed to its ability to modify the properties of the cell membrane lipid matrix that may lead to cell death [105,106,107]. Methanol is cytotoxic due to its inhibitory effect on cell proliferation at a concentration of more than 10% [108]. Complete removal can be achieved by ensuring the complete evaporation of residual solvents from the PHA pellets, considering the volatile nature of these solvents, followed by proper washing. Apart from chemical extraction, PHA can be extracted biologically by feeding PHA-harboring microbial cells to a mealworm species (*Tenebrio molitor*) that discharges PHA as waste [109].

Microbial production of PHA involves mainly Gram-negative bacteria, with *Cupriavidus necator* (also known as *Ralstonia eutropha*) as the standard PHA-producing bacteria among them. The endotoxins constitute lipopolysaccharides (LPS), heat-resistant components located in the outer cell membrane of Gram-negative bacteria, which are responsible for inflammatory reactions in biomedical applications of biomaterials. LPS are liberated during PHA extraction steps, where the cell biomass is lysed. The LPS then contaminates the resulting PHA and are carried along to the precipitation step, thus remaining on the resulting PHA pellets [104,110,111,112]. The proper and efficient removal of endotoxins is necessary to assure in vivo applicability, and common removal methods include using sodium hydroxide or hydrogen peroxide [113,114]. Alternatively, endotoxin-free PHA can be obtained by using Gram-positive bacteria for production instead, owing to the inability of Gram-positive bacteria to produce LPS. *Bacillus* is a popular choice, given its promising PHA yield and less stringent requirements for fermentation conditions [115,116].

Although PHAs are biocompatible, some of their physicochemical properties hinder wider use in several applications. Poly(3-hydroxybutyrate) [P(3HB)] and Poly(3-hydroxybutyrate-*co*-3-hydroxyvalerate) [P(3HB-*co*-3HV)] are the most studied PHAs for numerous biomedical applications [25,117,118,119,120]. The employment of homopolymer P(3HB) as a drug delivery device is discouraging due to its inherently poor thermal stability that leads to limited processibility and uncontrollable drug release kinetics [121]. The incorporation of a 3-hydroxyvalerate (3HV) monomer provides better flexibility and strength, with reduced chain packing and toughness in the resulting copolymeric P(3HB-*co*-3HV) [122,123,124,125,126]. P(3HB-*co*-3HV) is attractive as a drug delivery agent, as the higher molar fraction of 3HV in the copolymer contributes to a more amorphous structure that favors drug release [127,128]. Additionally, the incorporation of a second or even third monomer to form a copolymer or terpolymer also leads to lower crystallinity which is a desirable characteristic for PHA-based implants that require sufficient biodegradability [129]. In addition to biodegradability, enhanced mechanical properties can also be achieved to provide support in arteries as a stent in angioplasty [130]. However, PHAs have poor compatibility with therapeutic agents, which results in a low encapsulation efficiency due to their hydrophobicity, as mentioned earlier. Incorporation of hydrophilicity is generally done by functionalizing PHA with polar functional groups, or by block/graft copolymerization of PHA with hydrophilic components such as poly(ethylene glycol) [49,131].

Despite the excellent potential of PHAs, their high selling price is a major drawback when considering their use in medical applications. As the carbon sources employed in PHA manufacturing contribute to 30–40% of the overall production costs, the employment of waste in substitution of defined carbon sources was attempted to make PHA more affordable and economically competitive [132,133]. With increasing emphasis on sustainability, waste plant oils are gaining great interest [134]. One concern in using such PHAs for biomedical applications is that the residual oils will adhere to bacterial biomass, then be carried forward to subsequent downstream processing. Residual oil removal is usually carried out using non-polar organic solvents such as hexane, which has a polarity index of 0.1. The cell biomass must be washed repeatedly with clean water after primary washing with solvents for complete removal [135,136]. Alternately, residual oil removal can also be done using supercritical CO_2_ and CO_2_-expanded ethanol. The supercritical CO_2_ method has been shown to remove impurities of more than 70 wt% from P(3HB). In contrast, more than 93 wt% of residual oils were removed by adding a small volume of ethanol in the presence of CO_2_, which lowered the pressure requirement for the oil removal process [137]. As CO_2_ and ethanol are easily recyclable and relatively harmless, the employment of organic and hazardous solvents can be minimized.

Since the discovery of PHAs in 1888 by Martinus W. Beijerinck, the polymers have been extensively studied for the last eight decades due to academic interest [138]. The introduction of eco-friendly bio-based products as alternatives to conventional plastics brought the term ‘bioplastics’ to public attention. Although PHAs have generated a lot of interest in the last few decades due to their material properties, biocompatibility, and sustainability, the selling price of PHAs is still the main determining factor for commercialization [139]. The development of PHA production strategies is a continuous effort to bring about a higher PHA yield and economic attraction. Designing, evaluating, and optimizing PHA-based formulations for pharmaceutical and therapeutic applications still require more studies before their wide implementation in pharmaceutical industries due to the disadvantages resulting from their material properties [140,141]. Although there is still much to be explored, emerging knowledge is turning PHAs into convenient, high-performance, and economically competitive polymers that can be widely accepted and implemented for medication in the foreseeable future.

## 5. Conclusions

PHAs are an outstanding group of bioplastics renowned for their biocompatibility, biodegradability, ease of modification, ability to encapsulate drugs, as well their impressive pharmacokinetics and pharmacodynamics that surpass those of free drug therapy or other polymeric nanocarriers. PHA nanocarriers are also superior in the sense that they can degrade naturally into components that are nontoxic to the human body. PHA nanocarriers can be formulated using a wide range of techniques, such as the immensely popular emulsion solvent evaporation methods (oil-in-water, single emulsion, and water-in-oil-in-water double emulsion), nanoprecipitation, dialysis, and in situ polymerization. PHA nanocarriers are commonly utilized in tissue engineering applications and even in fighting infections, with antibiotics being incorporated into PHA nanocarriers to improve the effectiveness of treatment. In cancer therapy, PHA nanocarriers are advantageous because the hydrophobic nature of the polymer aids in a higher drug load and a slow, controlled drug release.

Unfortunately, the hydrophobicity of PHA can be undesirable when it limits solubility, but this is often countered by functionalization with hydrophilic substances. In a similar way, copolymerization and terpolymerization have been convenient solutions to combat certain PHAs’ poor physical properties like thermal instability. Though PHAs themselves are non-immunogenic, certain environmentally unfriendly organic solvents used during their production, such as chloroform and methanol, can contribute to their cytotoxicity. A remedy for this would be proper washing and endotoxin removal steps or opting out of said chemicals in favor of biological extraction methods. Another major disadvantage of PHAs lies in their high production costs; however, using waste materials as carbon sources tends to lower such costs significantly.

All in all, despite the stagnation of PHA nanocarriers in clinical trials of drug delivery applications, they are undoubtedly a move in the right direction. Though many solutions have been discovered to overcome obstacles in their path, further research will always lend a helping hand towards the promisingly bright future of PHA nanocarriers in effective drug delivery systems.

## Data Availability

Data sharing is not applicable for this review.
